# The Cambridge Sympathy Test: Self-reported sympathy and distress in autism

**DOI:** 10.1371/journal.pone.0198273

**Published:** 2018-07-27

**Authors:** Rosemary Holt, Jessica Upadhyay, Paula Smith, Carrie Allison, Simon Baron-Cohen, Bhismadev Chakrabarti

**Affiliations:** 1 Autism Research Centre, Department of Psychiatry, University of Cambridge, Cambridge, United Kingdom; 2 Exeter University Medical School, Exeter, United Kingdom; 3 Centre for Autism, School of Psychology & Clinical Language Sciences, University of Reading, Reading, United Kingdom; Universite de Bretagne Occidentale, FRANCE

## Abstract

**Background:**

Difficulties with aspects of social interaction, including empathy, comprise a core symptom of autism spectrum conditions (autism). Sympathy is a specific form of empathy and involves both cognitive and affective empathy. Data are presented from a new task of self-reported sympathy and personal distress.

**Methods:**

Participants with autism (93 males; 161 females) and controls (40 males, 93 females) took part in an online survey via the Autism Research Centre or Cambridge Psychology websites. Participants completed a task where they were asked to rate photographic images that were either of distressing, neutral or happy scenes, according to the amount of sympathy they had for the individual in the photo and the degree of personal distress they felt. All participants also completed the Empathy Quotient (EQ).

**Results:**

Significant differences were found between the autism and control groups for both self-reported sympathy and personal distress, with participants with autism giving lower ratings than controls. Control females scored significantly higher than control males in both sympathy and distress. Sympathy and distress ratings in the autism group did not differ significantly by sex. EQ showed positive correlations with sympathy and distress scores.

**Conclusions:**

Using a new measure of self-reported sympathy, we found that both males and females with autism gave lower ratings of sympathy when viewing people in distressing scenarios, compared to controls.

## Introduction

Autism is a spectrum of neurodevelopmental conditions, characterised by difficulties in reciprocal social interaction and communication, difficulties in adjusting to unexpected change, as well as the presence of unusually narrow interests and repetitive behaviours, and sensory hypersensitivity (DSM-5, 2013). Previous studies have examined empathy and theory of mind (also called mentalizing, or cognitive empathy) in autistic individuals, finding below average performance [1–5]. However, the different aspects of empathy and how these might differ in autistic individuals may be more complex, with evidence suggesting that impairments may apply to some aspects but not others.

Empathy involves understanding and responding to the direct, perceived imagined or inferred feeling or state of another being [4, 6, 7, 8, 9]. It can be further fractionated into cognitive empathy, affective empathy, and sympathy [10, 11]. Cognitive empathy involves inferential processes in order to attribute mental states to oneself or others. This involves understanding the thoughts, feelings and intentions of others and using such mental state information to predict that person’s behaviour. In contrast, bottom-up processes lead to an emotional response in the observer to another person’s emotional state. This fraction includes the phenomenon of emotional contagion, i.e when one person’s emotion triggers an emotional state in the observer but does not necessarily involve understanding the other’s emotion. (An example is when one baby cries and this may trigger a second baby to cry). Affective empathy could therefore lead one to feel distressed on seeing others in distress, and lead to ‘personal distress’ (PD). PD can in turn lead to the desire to alleviate one’s own negative valenced state, which might manifest in an aversive reaction to other’s distress [12]. In contrast, sympathy is focused on the other person’s distress and involves a ‘concern mechanism’ [10]. It involves recognizing the sadness or suffering of others, and responding to this with an emotion, such as feeling sorrow or pity, and a desire to alleviate their suffering [13].

Sympathy may involve elements of both cognitive and affective empathy, but is independent from these concepts. Empirical reports support this theoretical distinction by showing that sympathy and personal distress are differentially associated with prosocial behavior, with sympathy being positively associated and distress being negatively related [14,12].

The dissociation between different components of empathy is further illustrated in clinical conditions where cognitive and affective empathy are differentially affected. For example, in autism, affective empathy may be intact but cognitive empathy is impaired, whilst the opposite pattern has been suggested for patients with conduct disorder [15] or psychopathy [16, 17]. The results from case-control studies is further supported by evidence from the general population using trait measures; high psychopathic traits are associated with reduced affective resonance, whereas high autistic traits are associated with reduced perspective taking [18]. Other evidence suggests an association between high autistic traits and reduced prosocial behaviour in the general population [19].

Previous research examining empathy found autistic individuals had difficulty identifying other’s mental states from images of people, but did not differ from controls in reports of their own emotional response [20]. Rueda et al [21] identified intact empathic concern and personal distress but lower scores on measures of cognitive empathy in young people with Asperger syndrome, compared to controls. A psychophysiological study showed comparable electrodermal responses to distressing stimuli in autistic and neurotypical children [22]. Although few studies have looked specifically at self-reported sympathy in autistic individuals, research suggests that this capacity may be intact [23]. However, self-report measures of personal distress have shown a difference between typical and autistic adults in self-reported mood ratings to emotionally distressing stimuli. These responses were also positively correlated with empathy as measured by the Empathy Quotient (EQ) [24].

Sex differences in empathy (females on average scoring higher than males) have been identified in the general population [25], and these are reduced or attenuated in autistic people, [1, 4, 5, 26–28] in line with the ‘extreme male brain’ theory [29]. Despite the many studies investigating empathy in people on the autistic spectrum, few have focused on sympathy. Age related findings have also been reported in typical populations, with more extreme responses with increasing age [30] and higher levels of emotional and cognitive empathy with age [31]. To our knowledge comparable investigations of empathy and age have not been investigated in autistic individuals.

The Cambridge Sympathy Task was designed to address this gap in the literature. Specifically, it was designed to examine self-reported levels of sympathy and personal distress in response to distressing emotionally charged vs. neutral or happy images. The aims of the present study were first, to test for group differences on the task between neurotypical and autistic individuals. Secondly, we examined sex differences within both groups. Finally, we tested if there is a significant relationship between reported sympathy or personal distress levels with self-reported trait empathy as measured by the EQ and examined associations between task responses and age.

## Materials and methods

### Participants and ethics information

The autism and control groups were age and sex-matched. The final sample comprised n = 387 participants: 93 males with autism aged 25–69 years, 161 females with autism aged 16–65 years, 40 neurotypical males aged 22–67 years and 93 neurotypical females aged 16–65 years. In total, 711 participants recruited from the Cambridge Autism Research Database (CARD) took the Cambridge Sympathy Test by logging in at the Autism Research Centre (ARC) website (www.autismresearchcentre.com) or at a linked website for those without a diagnosis (www.cambridgepsychology.com). Participants consented to participate in the research and for their data to be stored in the CARD. Ethical approval was granted by the University of Cambridge Psychology Research Ethics Committee (reference number Pre.2013.06).

The autism group included those with a self-reported clinical diagnosis of autism, participants were asked for information regarding the date of their diagnosis, where they received their diagnosis and the professional that diagnosed them. All participants also completed the Autism Spectrum Quotient (AQ) [32], as a meaure of autistic triats in both groups and to ensure that the control group were not scoring higher that expected on this questionnaire.

The control group were recruited through www.cambridgepsychology.com. Individuals reported that they did not have a diagnosis of autism, or any first-degree family member with autism. Scores on the AQ were used to ensure this group was comparable to the general population in terms of autistic traits [33]. A random sample of 6% of typical males and 2% of typical females who scored >26 on the AQ were included, and the remainder of those above this threshold were excluded. Based on the AQ scores identified in the broader autism phenotype [34], this methodology has been used in previous research to avoid a biased sample of high scorers on the AQ in the control group where online collection methods are used [35,36].

### Task development

Prior to the study, the stimuli were validated to ensure that the control and sympathy evoking images scored different ratings for evoked sympathy and personal distress. 40 control participants recruited from within and around the University of Cambridge (18 typical males and 22 typical females) aged 18–21 years, completed the task in a lab setting. The two types of images (distressing vs. other) evoked significantly different responses, with the distressing images resulting in higher self-reported sympathy (t = -31.106, df = 54.94 p<0.05) and personal distress scores (t = -28.44, df = 45.82, p<0.05). These control data also indicated a significant sex difference for sympathy ratings (t = 2.721, df = 38, p < .01), with females on average scoring higher on this measure.

### Stimuli

The task stimuli involved 80 black and white images, 40 of which were distressing and considered ‘sympathy evoking’, and the other 40 were control images of neutral or happy scenarios. Images were presented twice, and the responder was asked to rate the images for self-reported levels of sympathy and personal distress, separately. In the sympathy block, participants were asked to rate the images on a scale of 1 (“I feel no sympathy for the person/s in the photo”) to 6 (“I feel a lot of sympathy for the person/s in the photo”). During the distress block participants were instructed to rate their *own* emotion on a scale of 1 (“I do not feel sad at all”) to 6 (“I feel very sad”). Emotionally distressing images included scenes such as devastation from an earthquake, a domestic abuse victim, or child labour. In contrast, non-distressing control images included scenes such as washing fruit, baseball practice and reading the paper. Each image contained a human face and was accompanied by a brief phrase describing the scene. The images were presented in the same order for each participant. Stimuli were presented for 5 seconds and the inter stimulus interval was 1 second. The sympathy and distress blocks were counterbalanced.

### Procedure

**Fig 1:**
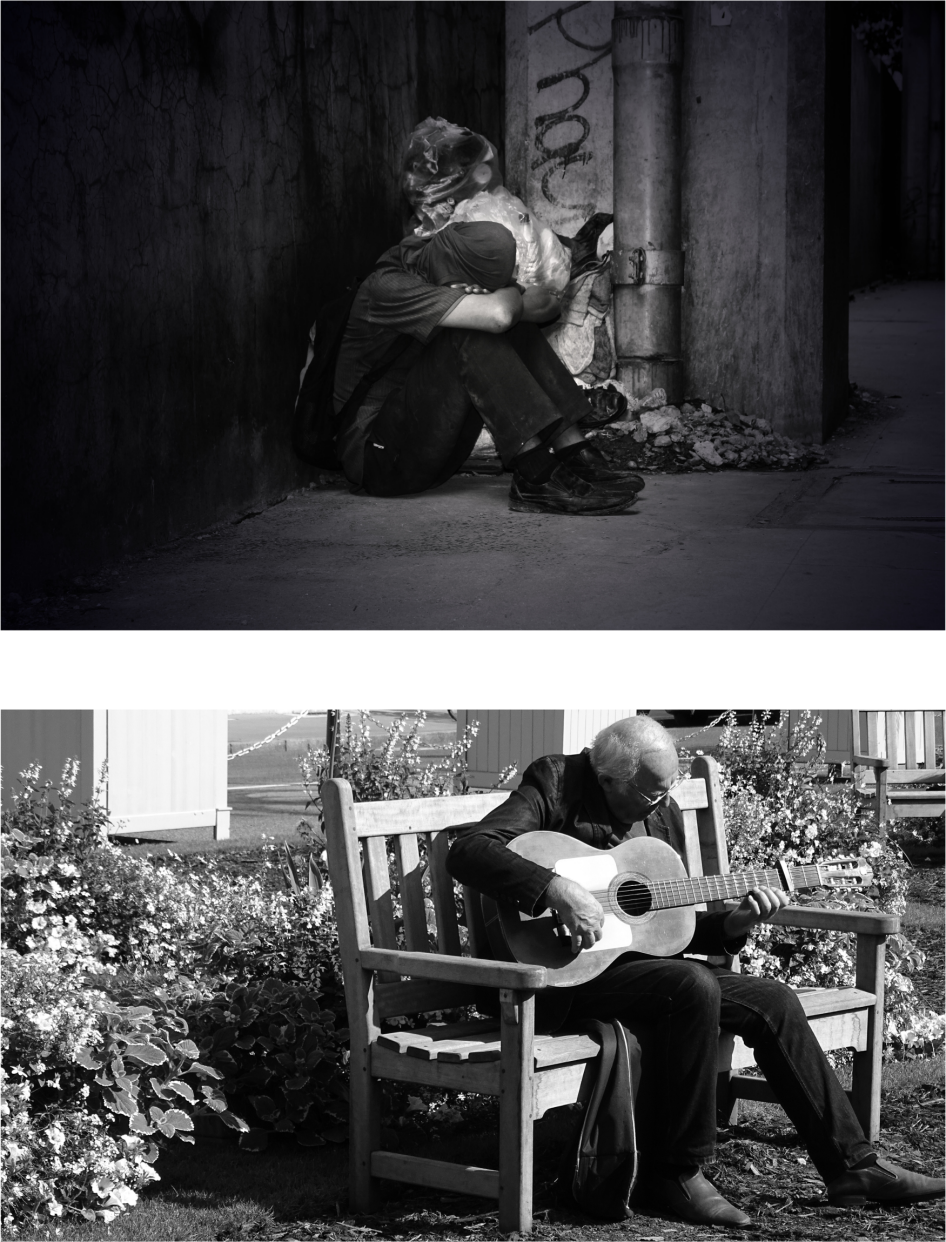
Upper image, example of distressing image. Lower image, example of a non-distressing image. Representative images are used in accordance with the copyright restrictions of the original images (photo credit: Pexels.com). Stimuli from the task are available on request.

The Cambridge Sympathy Test was administered online (Representative images shown in Fig 1). Participants completed it in their own time on their own computer. Instructions were given at the beginning of each block informing participants they would see a series of photographs of people in various real-life situations. Participants were instructed to rate their sympathy and distress on a scale of 1–6, depending on which block was presented to them first. For those who carried out the task more than once, only their first trial responses were counted. Participants were also invited to complete the EQ [4].

### Data analysis

Data analyses were planned a-priori to examine group and sex differences on the task and the association with EQ scores. A Chi-Square test was performed pre-and post-gender matching to test if the sex ratios were matched in the two groups. The groups were also matched on age. A Shapiro-Wilk test was performed to test deviations from normality in the data. Since both sympathy and personal distress rating data showed significant deviations from normality, appropriate non-parametric statistics were used. Mann-Whitney U tests were used to identify if the case and control groups differed in their self-reported sympathy and personal distress scores in response to distressing and non-distressing images. Sex differences within the autism and control groups were examined in addition to sex-stratified analyses comparing cases and controls. Spearman's Rank Order Correlation test was used to determine a relationship between sympathy and distress mean scores. Correlations were performed to examine the relationship between EQ and sympathy and personal distress ratings. Lastly, correlation analyses were conducted on the association between age and sympathy and personal distress ratings. See Table 1 for means, standard deviations of the sample demographics.

**Table 1:** Demographic characteristics of the sample including means and standard deviations.

	Autism Mean	Standard deviation	Control Mean	Standard deviation	Group difference
Sex ratio (M:F)	93:161		40:93		X^2^(1) = 1.65 p = .198
Age (years)	41.14	12.16	41.25	12.27	t = -.081 p = .935
AQ	36.95	9.51	17.20	6.86	t = 23.206 p < .001
EQ	21.31	14.76	46.57	15.03	t = -15.45 p < .001
Sympathy mean	4.43	1.29	5.16	.62	U = 11435 p < .001
Personal distress mean	3.75	1.51	4.69	.89	U = 11006.5 p < .001

## Results

### Group differences

Significant differences were found between the autism and control groups for ratings of sympathy (U = 11435, p < .001), and personal distress (U = 11006.5, p < .001), with autistic participants giving lower ratings than controls. There were no significant group differences in the response to the control images. When stratifying by sex there was a significant difference between autistic and control females on both sympathy (U = 4755.5, p < .001) and personal distress ratings (U = 4336, p < .001), with autistic females scoring lower than control females.

**Fig 2:**
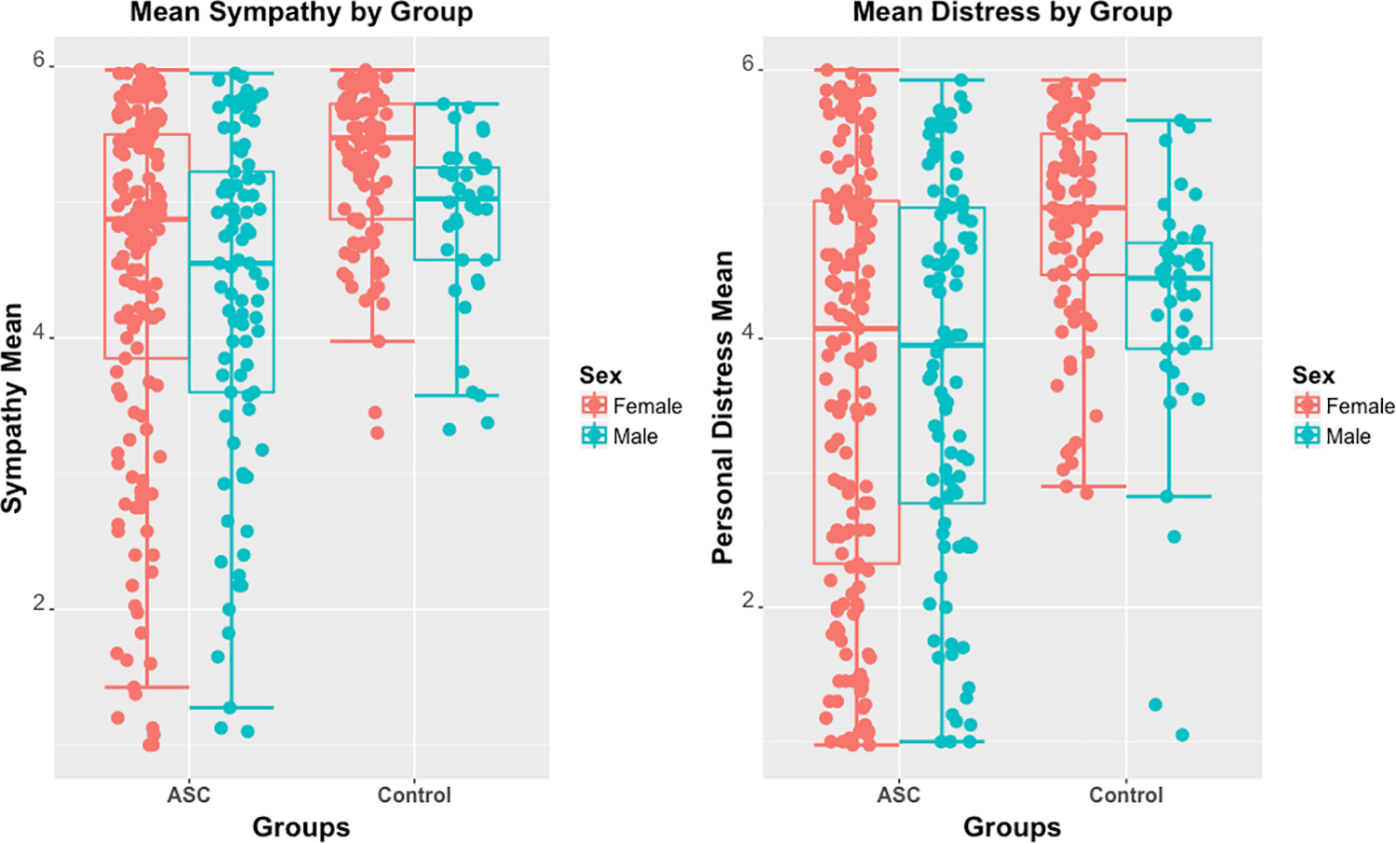
Plots illustrating sympathy and distress means +/- 2 SE for autism and control groups stratified by sex.

Autistic males gave significantly lower sympathy ratings than control males (U = 1438.5, p = .039) but there was no significant difference between autistic and control males for personal distress ratings (U = 1573.5, p = .160) (see Fig 2 for plots of group differences).

### Sex differences

Control females scored significantly higher than control males on both sympathy (U = 1043, p < .001) and personal distress (U = 974, p < .001). Sympathy and personal distress ratings did not differ significantly by sex in the autism group (Sympathy: U = 6663.5, p = .145, Personal distress: U = 7423, p = .910). There were no significant sex differences in ratings of the control images.

### Empathy quotient

There was a statistically significant difference (t = -15.45, p < 0.001) between the autism and control group on EQ scores. Results indicated that autistic males (mean = 17.63, SD = 11.12) and autistic females (mean = 23.36, SD = 16.116) scored lower on the EQ than their sex-matched controls, (mean = 39.58, SD = 13.52 for typical males, and mean = 49.52, SD = 14.72 for typical females). In both groups, females scored significantly higher than males (t = -4.55, P < .001).

### Correlations

**Fig 3:**
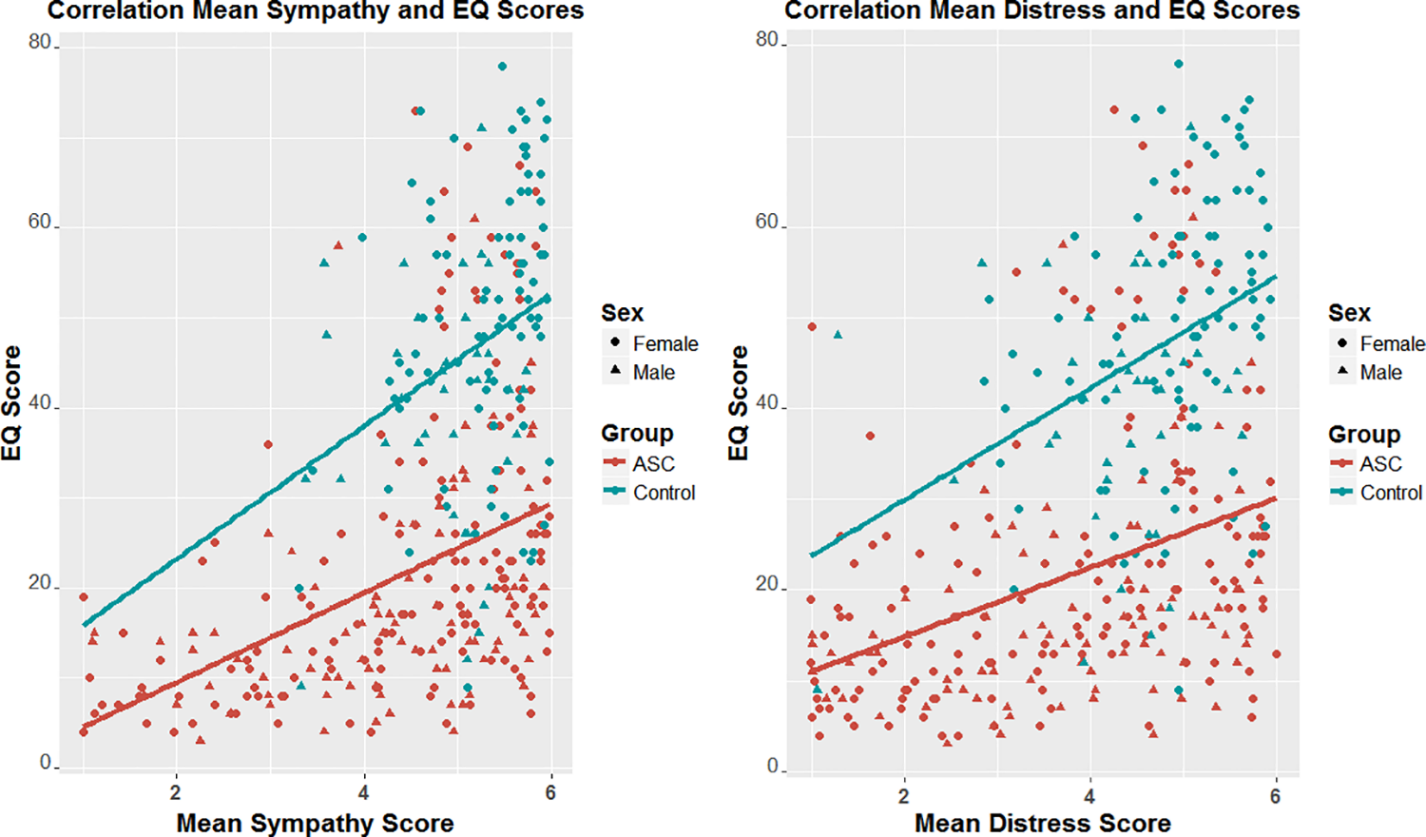
Left panel: Scatterplot showing the association between EQ and self-reported sympathy ratings; Right panel: Scatterplot of association between EQ and personal distress ratings. Scores from the autism group are shown in blue. Scores from the control group are shown in green. A line of best fit is shown.

There was a significant correlation between mean self-reported scores for sympathy and personal distress (r = .812, p<0.001). There was also a significant positive correlation between EQ scores with sympathy (r = .502, p<0.05) and personal distress (r = .494, p<0.001) ratings across the whole sample. There was a significant correlation between EQ scores with sympathy (r = .332, p<0.001) and personal distress (r = .403, p<0.001) in the control group. In the autism group this correlation was also evident with both sympathy (r = .519, p<0.001) and personal distress ratings (r = .452, p<0.001) (see Fig 3). There was a small but significant group difference in the strength of correlation between EQ and sympathy (z = 2.08, p = 0.0375), with a stronger correlation seen in the autism group. There was no group difference in the association between EQ and personal distress. There was no correlation between age and sympathy (r = -.033, p = .597) and personal distress (r = -.005, p = .94) responses in the autism group. However there was a small correlation between age and sympathy (r = .206, p = .017) and personal distress (r = .229, p = .008) in the control group. The relationship with age differed significantly between the groups for sympathy (z = -2.24, p = .025) and personal distress responses (z = -2.2, p = .028).

## Discussion

This study reports a new measure, the Cambridge Sympathy Test, to assess self-reported sympathy and personal distress in autistic and neurotypical individuals. Autistic males and females gave lower sympathy ratings when viewing people in distressing scenarios, compared to controls. Autistic females reported lower levels of personal distress compared to neurotypical females. These results are in line with previous research demonstrating reduced self-reported empathy in individuals with autism [3,4, 26].

However, group differences found in personal distress ratings are inconsistent with some previous reports of an absence of a difference in reports of one’s own emotional response in those with autism on a similar task [20]. This may be due to design differences between the two studies and that autistic individuals perhaps interpret this task in a different way. Other factors in the data may also account for the differences such as one’s own emotional state [37]. These results should not be taken to indicate that people with autism are uncaring, as there is considerable evidence that they do care [23]; only that their processing of emotional cues in distressing scenes does not elicit the same level of self-reported sympathy or personal distress.

In this study, there was a significant positive correlation between sympathy and personal distress scores, suggesting a strong overlap between these aspects of empathy. When analysing sex differences on this task, results showed that on average, women gave ratings indicating higher levels of sympathy and personal distress towards distressing images compared to males. This is in keeping with the literature suggesting that on average typical females score higher on empathy measures, compared to typical males [38]. There was an absence of the typical sex difference on this task in the autism group, replicating the pattern of results seen on other measures of empathy [4, 36, 28].

The range of self-reported ratings on this task was much broader in the autism group, suggesting a more heterogeneous response to this task (as can been seen in Fig 2). This is consistent with the literature demonstrating heterogeneity in autism [39, 40]. This also suggests that there may be subgroups of autistic individuals that respond differently to this task and this may be characteristic of other social-cognitive differences. Future work could investigate this hypothesis with unsupervised data-driven stratification approaches [41]. Task ratings were correlated with self-reported scores on the Empathy Quotient, providing validation for this task and demonstrating an association between self-reported ratings of sympathy and personal distress and traits of empathy. This points to some overlap in these concepts of around 50% which is consistent with previous theoretical description on overlapping but dissociable concepts [10]. The strength of correlation between sympathy and EQ was significantly stronger in the autism group suggesting that these traits are more closely linked in autistic individuals. However as mentioned previously the range of sympathy ratings was broader in the autism group which may account for this difference. There was no difference between groups in the strength of association between empathy traits and personal distress ratings suggesting that empathy scores are equally related to personal distress irrespective of autism diagnosis. There was a strong association identified between sympathy and personal distress responses on this task suggesting that these components of empathy may be closely linked. As seen in previous studies, scores on the EQ in this cohort were significantly lower in the autism group compared to controls [2, 4, 5]. An increase in sympathy and personal distress ratings with age in neurotypical individuals was observed which was not seen in autistic individuals. This unexpected finding merits further follow up ideally with longitudinal studies of individuals with and without autism. This new test could be a useful tool in assessing sympathy in autistic individuals as it is less dependent on language compared to some other measures [3].

### Limitations

The study has several limitations. First, this task was administered online, and so we were not able to control for device used, environmental distractors, or check for participant understanding of task instructions. Verbal ability was not measured in this study. Although this task is less reliant on verbal skills it could still have had an impact on task performance. Future work should rule out any association with verbal IQ. Second, we did not exclude participants with other psychiatric or neurological conditions, which may have affected performance on the task. Third, as the Cambridge Sympathy Test is a self-report measure, it could be subject to unintentional bias. Behavioural and implicit measures of sympathy would be useful to validate the current findings. Finally, the context of many of the images portrayed in the scenes are not often seen first-hand in the UK and USA where most of the responders were based. This could have disproportionally impacted on the responding of autistic individuals due to the imagination required to respond about these less familiar contexts. Future work including more familiar scenarios would be helpful in determining if this factor influenced the results in the current study.

### Future directions

Cross-cultural differences in understanding and experience of sympathy are important considerations for future research [42, 43]. Further investigation is also required to replicate the current findings and to determine whether differential responding is in response to the cognitive or affective empathy aspects of the task. Future work could incorporate measures of physiological arousal (e.g., GSR, EMG) in response to distressing scenarios, as a physiological index of how people with autism differ in their emotional response. Incorporating physiological response could be important for understanding aspects of emotional empathy, [44–46]. In a recent study, Trimmer et al. [24] identified comparable physiological responses, but an impairment in the ability to interpret this response in an autism group compared to controls, a result that would be consistent with an early report [22]. We would predict that the physiological response to the sympathy task in individuals with autism may be comparable to that of controls. This task could also be adapted for use in functional brain imaging studies to investigate the neural correlates of the processing and response of sympathy in autistic individuals. Evidence from previous studies indicates a different neural response in autism during tasks of emotion recognition and theory of mind [47]. Future work could also compare data from this task to that of theory of mind tasks such as the ‘Reading the Mind in the Eyes’ task [3], the Multifaceted Empathy Task [20] or other facial emotion recognition tasks. These measures may be associated with performance on the Cambridge Sympathy Test, and a combination of such tasks could aid in the identification of social-cognitive profiles in autism and other conditions where empathy may be affected such as Conduct Disorder and Psychopathy [15, 16]. This may assist in the design of more targeted therapies for these groups of individuals. Future research could investigate the presence of different subgroups based on performance on such a combination of tasks, and clinical traits associated with these subgroups. The relationship between responding on this task and pro-social behavior, in groups of individuals with and without autism, would also be of interest for future research, as the relationship between empathy and prosocial behaviour has been demonstrated in previous research [14]. Finally, although reaction times were recorded they were not analysed in this study due to device variability that was not recorded (Windows, iOS devices, etc.,), thus introducing a large source of uncontrolled variance in the data. Future work could consider if group difference in reaction times are present.

## Conclusions

This study reports data from a novel task of self-reported sympathy and personal distress to provide further insights into group differences seen in autistic individuals. In this study autistic individuals scored lower on sympathy and personal distress ratings compared to controls. This should not be taken to mean that people with autism have less care towards others’ suffering, and previous research shows people with autism are not like those with antisocial personality disorder who care less about others’ suffering [48]. Rather, differences on this task between those with autism and typical controls likely reflect reduced cognitive (but not affective) empathy, as well as difficulties with self-reporting of emotions. Also, typical women on average gave significantly higher ratings than typical men on sympathy and personal distress, whilst sex differences were not observed in the autism group. Positive correlations were identified between both sympathy and personal distress ratings with EQ, providing further validation of this measure. The Cambridge Sympathy Test is recommended for research into this specific aspect of empathy (sympathy) in both clinical and non-clinical populations, and has implications for approaches to therapy and education.
